# Separating vascular and neuronal effects of age on fMRI BOLD signals

**DOI:** 10.1098/rstb.2019.0631

**Published:** 2020-11-16

**Authors:** Kamen A. Tsvetanov, Richard N. A. Henson, James B. Rowe

**Affiliations:** 1 Department of Clinical Neurosciences, University of Cambridge, Cambridge CB2 0SZ, UK; 2 Department of Psychology, University of Cambridge, Cambridge CB2 3EB, UK; 3 Department of Psychiatry, University of Cambridge, Cambridge CB2 0SP, UK; 4 Medical Research Council Cognition and Brain Sciences Unit, University of Cambridge, Cambridge CB2 7EF, UK

**Keywords:** neurovascular, cerebrovascular, cardiovascular, ageing, fMRI, cognitive function

## Abstract

Accurate identification of brain function is necessary to understand the neurobiology of cognitive ageing, and thereby promote well-being across the lifespan. A common tool used to investigate neurocognitive ageing is functional magnetic resonance imaging (fMRI). However, although fMRI data are often interpreted in terms of neuronal activity, the blood oxygenation level-dependent (BOLD) signal measured by fMRI includes contributions of both vascular and neuronal factors, which change differentially with age. While some studies investigate vascular ageing factors, the results of these studies are not well known within the field of neurocognitive ageing and therefore vascular confounds in neurocognitive fMRI studies are common. Despite over 10 000 BOLD-fMRI papers on ageing, fewer than 20 have applied techniques to correct for vascular effects. However, neurovascular ageing is not only a confound in fMRI, but an important feature in its own right, to be assessed alongside measures of neuronal ageing. We review current approaches to dissociate neuronal and vascular components of BOLD-fMRI of regional activity and functional connectivity. We highlight emerging evidence that vascular mechanisms in the brain do not simply control blood flow to support the metabolic needs of neurons, but form complex neurovascular interactions that influence neuronal function in health and disease.

This article is part of the theme issue ‘Key relationships between non-invasive functional neuroimaging and the underlying neuronal activity’.

## 1. Introduction

The worldwide population is rapidly ageing, creating a pressing need to understand the neurobiology of healthy cognitive ageing, over and above the problems associated with the rise of dementia in ageing societies [1]. Understanding the neural mechanisms of healthy ageing will inform efforts to maintain cognitive function, which is critical for well-being across the lifespan [2]. While neuroimaging has led to advances in knowledge about relationships between neural function and cognition, the effects of age on these interactions are poorly understood. This is owing in part to outdated methodology, inadequate awareness and treatment of confounding variables, opaque reporting of results, lack of replication and a failure to consider the limitations of the signals of interest. In this paper, we review two complementary disciplines, neurocognitive ageing and neurovascular ageing, which have suffered from these limitations and have proceeded somewhat independently. We argue for a better understanding of their relative contributions to functional magnetic resonance imaging (fMRI) signals, so as to formally integrate them in models of successful ageing, avoid common misinterpretations of fMRI and provide solutions to the limitations within each discipline alone.

**Figure 1.**
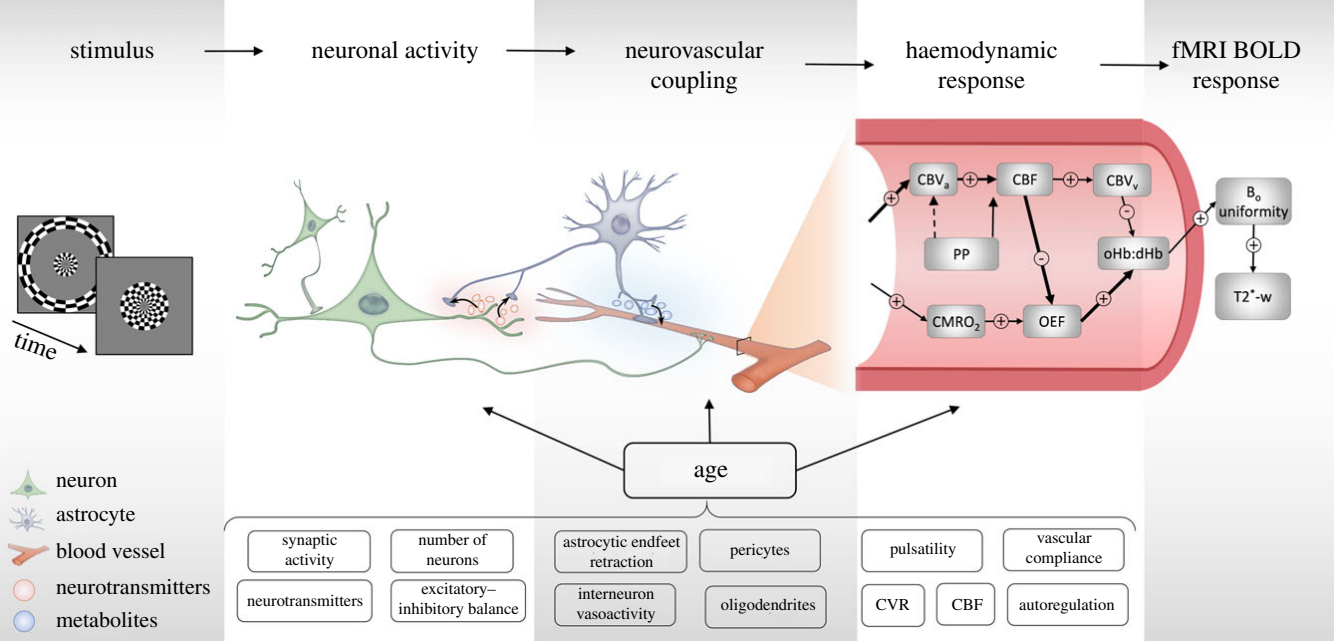
A schematic illustration of the physiological basis of the BOLD response. *Neuronal activity* elicited by a *stimulus* or background modulation gives rise to a complex *neurovascular coupling* signalling cascade. That triggers a *heamodynamic response* resulting in a blood-oxygen-level-depedent (BOLD) signal owing to changes in the magnetic field inhomogeneity detected as a T2*-weighted signal by an MRI scanner. (Lower panel) Some of the suspected mediators of the differential age effects on the processes that give rise to the BOLD response. CBV_a_, arterial cerebral blood volume; CBV_v_, venous cerebral blood volume; CBF, cerebal blood flow; CVR, cerebral vascular reactivity; CMRO_2_, cerebral metabolic rate of blood oxygen consumption; oHb, oxygenated heamoglobin; dHb, deoxygenated heamoglobin; OEF, oxygen extraction fraction; B_0_, magnetic field; PP, pulse pressure.

The literature on neurocognitive ageing over the past 30 years has extensively relied on the blood oxygenation level-dependent (BOLD) signal detected for most fMRI. The fMRI signal reflects changes in deoxyhaemoglobin concentrations in response to neural activity (figure 1). These concentrations change because increases in local synaptic activity and neuronal firing rates consume energy, which is sourced by transient local increase of cerebral blood flow (CBF) and cerebral blood volume (CBV). In simple terms, the dominant consequence is a temporary increase (‘over-compensation’) in oxygenated haemoglobin in the capillary and venous bed draining the activated region, reducing the concentration of deoxyhaemoglobin. Since deoxyhaemoglobin is paramagnetic, decreases in its concentration in turn increase the BOLD signal. The biophysical models of this *neurovascular coupling* include equations for dynamics of CBF, CBV and the cerebral metabolic rate of blood oxygen consumption (CMRO_2_; for more details see [3–5]). The resulting BOLD changes to a brief (less than 1 s) period of neuronal activity that can last up to 30 s, with a characteristic temporal profile that is known as the *haemodynamic response function* (HRF) [6–9]. Many of the processes represented by parameters in these biophysical models are affected by ageing, owing for example to age-related changes in vascular health. Therefore, a failure to consider changes in vascular health can mean that differences in fMRI signals are erroneously attributed to neuronal differences [10–12] and in turn their cognitive relevance is misunderstood [13–15].

In this review, we first consider some of the main mediators of the transformation of neural activity into a haemodynamic response. We show how age-related alterations in the neuro–vascular interaction can influence the interpretation of changes in BOLD signal. This leads to changes in the measurements of regional activity and connectivity. We then turn to emerging evidence for the complex physiological changes with age, which give rise to slowing of cognitive function. These motivate the development of new models that characterize the joint contribution of vascular and neuronal influences to fMRI, in order to better understand the neurobiology of cognitive ageing. The continued interest in fMRI, above methods that are not affected by the vascular effects such as magneto- or electro-encephalography, rests on its safety, wide availability, high spatial resolution and full brain depth of imaging.

## 2. Age-related changes in neuro–vascular influences

The study of neurovascular pathology has been relevant to understanding many medical, neurological and psychiatric disorders. Alterations in the neurovascular system during healthy ageing have also been studied at both the cellular and structural levels. These changes typically remain undiagnosed and may have no directly apparent consequences for cognitive function, but they may compromise vasculature and the ‘neurovascular unit’ that couple neuronal activity to vascular responses. This undermines the straightforward interpretation of BOLD as an index of neurometabolic activity in older populations, those on drugs that influence vascular function and many diseases that alter the neurovascular unit. The following section reviews the major neurovascular changes related to ageing, and considers the physiological consequences of structural changes for the BOLD fMRI signal.

### (a) Cellular and structural/morphological changes

#### (i) Vasculature, blood vessels and the cerebrovascular tree

Age leads to alterations in the cerebrovascular ‘tree’ at molecular, cellular and structural levels [16–19]. Large elastic arteries dilate, stiffen and become atheromatous and tortuous, while the intima of the muscular arteries thickens [20]. Vascular stiffening is also associated with alterations in smooth muscle cells [21], calcification and disruptions in the collagen–elastin balance [22,23]. Some arterial alterations are coupled with capillary rarefaction, in addition to molecular and morphological changes that perturb the brain–blood barrier (BBB) [16,22,24]. Ageing is also associated with endothelial dysfunction, which contributes to dysregulation of vascular tone, astrocyte-dependent BBB permeability and nitric-oxide-dependent inflammation [19,25]. Damage to the endothelium aggravates vascular stiffening [22,26,27] and compromises the vessels' ability to dilate and constrict in response to variations of blood pressure or vasoactive substances [16,23]. In addition, there is an age-related impairment in the mechanism underlying electrical propagation of retrograde hyperpolarization signal along the endothelial cells, thereby impairing the remote vasodilation of upstream pial arterioles and increased perfusion in the capillary bed [25].

Pericytes are a group of microvascular mural cells embedded in the basement of blood microvessels that regulate blood flow both physiologically and pathologically [28] in addition to fine tuning vascular tone and BBB permeability with their contractile properties [29,30]. Age-related changes in pericytes [16,22] together with other mural cell alterations are likely to lead to changes in the vascular basis of the BOLD signal.

In short, disruption of a myriad of cerebrovascular factors acting on different levels of the vascular tree contributes synergistically to changes in neurovascular signalling, perfusion and reactivity.

#### (ii) Neuronal and non-neuronal cells

Neurons can directly control cerebral blood flow [31]. In particular, interneurons produce vasodilators [32,33] and vasoconstrictors [34], such as nitric oxide, prostanoids, endothelin etc. (for more information on vasoactive agents see [25,35]). Stimulation that selectively targets interneurons causes a relatively small increase in oxygen consumption but a relatively large increase in CBF. In contrast, stimulation of excitatory neurons causes relatively large increase in oxygen consumption but relatively small increase in CBF [36,37]. These results suggest that, while the primary driver of the BOLD response (i.e. CBF) is interneuron activation, additional CMRO_2_-mediated changes in BOLD signal reflect excitatory neuron-modulated oxygen consumption [31]. The interpretation of these findings in the context of ageing is important, given the dissociating effects of age on excitatory versus inhibitory signalling and synapses [38–41].

While the role of glial cells in the neurovascular unit is less well understood than neuronal and vascular components, increasing evidence implicates glial elements as mediators between neurons and blood vessels [42]. Astrocytes are a diverse population of glial cells whose functions include neurovascular signalling [43], linking neurons to their blood supply [44] and regulating the BBB [45]. Activated astrocytes release vasoactive agents via multiple signalling pathways at different levels of the vascular tree [46] independently from other endothelial pathways [47] including caveolea-mediated vasodilation in arterial endothelium [48]. Retraction of the astrocytic endfeet as part of the clasmatodendrotic response [49], together with changes in the immune response and calcium signalling, impairs by-product clearance as the BBB efficiency breaks down [16,50]. Age-related changes in glia [16,22] together with mural and endothelial cell alterations could be stronger than that of neurons [51] and are likely to lead to changes in the vascular basis of the BOLD signal. Future work needs to consider how glial elements could be measured, integrated with *in vivo* neuroimaging and accounted for in physiological ageing models.

While both glia and neurons play a role in the vascular basis of the BOLD signal, their relative contribution to baseline (endogeneous) BOLD signal versus evoked (exogeneous, e.g. task-based) BOLD remains unclear. This is important because the baseline of blood flow, which decreases with ageing, can affect the sign and the magnitude of the evoked BOLD signal, without changes in underlying neural activity [52–55]. Age may differentiate the separate factors that regulate artery tone [31] versus evoked responses [36]. Taken together, it appears that subtypes of glia, mural cells, endothelium and inter-neurons control the BOLD signal, independent of the activity of the neighbouring excitatory neurons, and likely through multiple signalling mechanisms that contribute synergistically to vasodilation. Multiple neurovascular coupling (NVC) pathways acting on different levels of the vascular tree crucially depend on well-orchestrated interplay between different cell types of the neuro-glio-vascular unit, which may provide multiple safety mechanisms [46]. Ultimately, an integrated understanding of age effects on all components of the neuro-glio-vascular unit is required for a better understanding of the physiological basis of neurocognitive ageing, especially where inferences are drawn from fMRI.

### (b) Physiological changes

It is the effects of age on cerebrovascular function that render interpretation of age differences in the BOLD signal so challenging. Cerebrovascular function can be assessed by measuring: (i) resting CBF, (ii) CBF responses to changes in arterial CO_2_, referred to as cerebrovascular reactivity (CVR), (iii) CBF responses to changes in blood pressure, referred to as cerebral autoregulation, and (iv) CBF responses to changes in neural activation, referred to as NVC. Cerebrovascular alterations also include brain pulsatility and the cerebral metabolic rate of oxygen extraction. Below we review these changes based on the common range of physiological recordings (see also [16,25,56,57]).

#### (i) Resting cerebral blood flow

Decrease in global baseline CBF with age has been reported in early studies using transcranial Doppler ultrasonography [58], radiotracer techniques [56,59,60] and phase contrast imaging [61]. These changes are widespread across the cerebral cortex and the basal forebrain. The physiology underlying the CBF decrease in the aged brain is still debated [62]. The main candidates include primary causes of impaired vasoactivity and cardiovascular regulation of CBF during ageing, rather than the reduction in cardiac output [63]. CBF decline may also reflect the secondary effects of brain atrophy and reduction in neural activity as a shift towards lower metabolic demands, rather than primary changes in vasculature. The finding that changes in CBF can affect the sign and magnitude of the evoked BOLD signal without affecting underlying neural activity [52–54] is in line with the deoxyhaemoglobin-dilution model [64–66]. Therefore, the decline in the baseline CBF with ageing has implications for the interpretation of fMRI studies of ageing.

#### (ii) Cerebrovascular reactivity

Cerebrovascular reactivity (CVR) is informative about vascular health. CVR is distinct from resting CBF, as it measures the ability of cerebral arteries and arterioles to dynamically regulate blood supply through dilation or constriction. In particular, CVR reflects the CBF responses to changes in arterial CO_2_, whereby elevated partial pressure of arterial CO_2_ (hypercapnia) causes dilation of vascular smooth muscle, leading to regional increases in CBF, while reduced CO_2_ partial pressure (hypocapnia) causes vasoconstriction leading to regional decreases in CBF. Vascular sensitivity to CO_2_ is very marked in the cerebrovasculature [67] and is thought to depend on intra- and extracellular pH changes that modulate vascular smooth muscle tone [68–70]. Therefore, CVR is considered to be a more direct measure of vascular endothelium and smooth muscle function compared to baseline CBF. CO_2_ quantification in cerebrovasculature has used transcranial Doppler ultrasound [71], radiotracer techniques [72] and contrast imaging [73]. There is general agreement across multiple imaging techniques that changes in CBF relative to changes in CO_2_ partial pressure are similar between brain regions under hypercapnia, but not under hypocapnia [74]. Experimental modulation in CO_2_ partial pressure has been used to validate non-invasive perfusion techniques [75,76], as well as biophysical [77] and biochemical [78] aspects of cerebral vasodilation.

Global decline in CVR with age has been reported using transcranial Doppler ultrasound [58], radio tracer techniques [60,79,80] and phase contrast imaging [81]. Age-related differences in the response of regional CBF to CO_2_ inhalation have been reported using PET [82]. Reduction in hypercapnia-induced vasodilation in the cerebellum and insular cortex, as well as hypocapnia-induced vasoconstriction in the frontal cortex, has been observed in older adults, suggesting less effective vascular response in cerebral perforating arteries [82]. Likely causes for CVR changes are arterial stiffening [83] and compromised endothelial function in blood vessels [84], which lead to a decreased vascular response to match metabolic demands. In addition, white matter hyperintensities, a common MRI finding in ageing, are associated with reduced baseline CBF and reduced response to hypercapnia [85,86]. Compromised CVR will lead to a reduced dynamic range of the BOLD signal, having direct implications for task-based fMRI studies of ageing: even with the same levels of neural activation across age groups, lower CVR in the older group would lead to smaller amounts of vasodilation and therefore reduced evoked CBF, reduced decrease of deoxyhaemoglobin concentration and reduced BOLD signal. Without controlling for CVR differences, this would lead to an under-representation of neural responses in older individuals.

#### (iii) Pulsatility

Cyclic cardiac contractions that pump blood through the arterial system generate a pulsatile blood flow and concomitant pulsatile pressure experienced by vascular wall tissue. This pulsatile phenomenon is absorbed before it reaches pressure-sensitive cerebral capillaries, and maintenance of steady flow and pressure ensures exchange of nutrients and clearance by-products. The first line of defence to minimize the effect of flow and pressure pulsatility in the microcirculation is achieved by the highly elastic aorta and muscular arteries, e.g. the aorta–carotid interface. The distensibility mismatch in these vessels dampens the pulsatile energy projected distally, known as the Windkessel effect [87,88]. Arterial stiffening caused by imbalance in elastin–collagen in the load-bearing intima of the aorta and central elastic arteries alters arterial distensibility, translating into increased pulse wave velocity [89]. The change of pulse wave velocity with age alters the wave reflection properties at the aorta–carotid interface, resulting in less effective cushioning of pulsations in the arterial system, i.e. diminished Windkessel effect, and greater transmission of pulsatile energy into the cerebral microcirculation [56]. Increase of pulsatility in the proximal part of cerebrovasculature is further exacerbated with age increase in pulse pressure (increased difference between systolic and diastolic pressure). Transcranial Doppler ultrasound of major arteries entering the brain, together with phase contrast MRI of the whole brain, both point to an age-related increase in cerebral pulsatility [90,91]. These changes can potentially contribute to microvascular ischaemia and tissue damage that is seen in some MRI-derived measures [90,92,93]. These microvascular changes have in the past been considered as a benign feature of ageing, but may actually be a significant contributor to changes in neurocognitive function [94]. With regards to BOLD imaging, pulsatile blood flow not only leads to fluctuation in signal intensity in arteries, arterioles and other large vessels [95], but also an age-related increase in pulsatility deeper in microvasculature. This could have dramatic effects on the BOLD signal in the proximity of neuronal tissue, which has only recently been recognized as a potential confound of BOLD studies [12,96–100].

#### (iv) Cerebral autoregulation

The second line of defence for minimizing pressure fluctuations in brain microvasculature is cerebral autoregulation, referred to here as autoregulation, via the vessels' ability to dilate or constrict in response to systemic perfusion pressure changes [74]. This is complementary to CVR [101,102]. In particular, autoregulation constitutes the ability of the cerebral vasculature to maintain steady flow and pressure in the capillary bed during transient changes in arterial pressure or intracranial pressure. The myogenic response, which is intrinsic to the vascular smooth cells and a key mechanism to autoregulation, is impaired with ageing, especially under conditions of hypertension and increased pressure pulsatility [57]. Furthermore, impaired autoregulation precedes vascular damage in white matter [103] and relates to white matter hyperintensities [104] in animal and human studies, respectively.

The CVR and autoregulation adjustment of vascular resistance to varying arterial CO_2_ and pressure, respectively, is primarily modulated in large arteries and pial arterioles [74]. This suggests that BOLD-related measures targeting CVR and autoregulation may be sensitive to ageing effects in the proximal part of the vasculature, and less sensitive to independent changes in the distal part of the cerebral circulation and physiological factors therein. In other words, CVR and autoregulation may be less sensitive to mechanisms underlying retrograde intramural propagation of vascular signals, causing remote vasodilation of upstream pial arterioles, i.e. impairing mechanisms of communication within the neurovascular unit [47].

### (c) Drug effects

Older people are more likely to be taking medications, including medications for age-related chronic disorders such as high blood pressure, diabetes, blood clotting, arthritis or neurodegeneration. Ageing studies often do not explicitly address these potential confounds in the interpretation of their results, which potentially modifies their conclusions, especially since the drugs are likely to affect the cascade of signalling and vascular events that form the basis of the BOLD signal [105]. Approaches aimed at dissociating vascular from neuronal signals should seek to identify, characterize and control for the confounding effects of drugs in ageing studies.

## 3. Dissociating neuro–vascular influences in BOLD fMRI signal

In order to interpret fMRI data, one needs to understand the contributions of neuronal and vascular components of signal variance. Some studies of neurocognitive ageing attempt to bypass the impact of vascular influences through their inclusion criteria, e.g. excluding individuals with a history of hypertension, cardiovascular or neurological conditions. These are, however, categorical criteria that are insensitive to continuous variation in the population and unable to resolve the effects of undiagnosed/presymptomatic conditions, not to mention producing results that are potentially biased in not generalizing to the typical ageing person.

**Figure 2.**
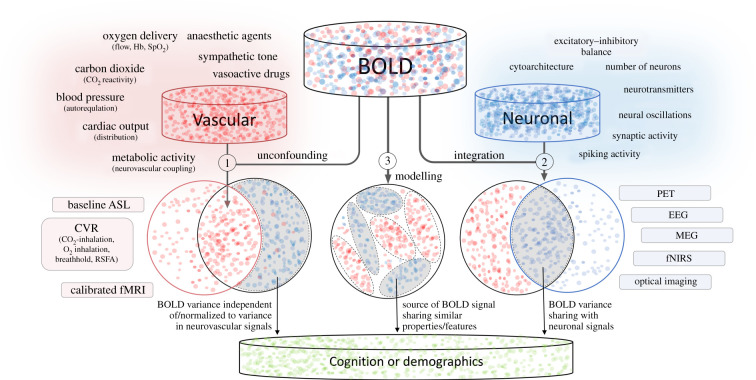
Schematic illustration of dissociating neurovascular influences in fMRI-BOLD using (1) vascular unconfounding, (2) neuronal integration and (3) BOLD modelling. ASL, arterial-spin labelling; EEG, electroencephalography; fNIRS, functional near‐infrared spectroscopy; MEG, magnetoencephalography; PET, positron emission tomography; RSFA, resting state fluctuation amplitude.

There are several other approaches to separate, or ‘unconfound’, neural from vascular factors. These are summarized as three broad strategies (figure 2). The first is based on detecting vascular signals in BOLD fMRI data by using independent measurement of vascular signals, termed here *vascular unconfounding*. The second relies on identifying neuronal signals in BOLD fMRI by using independent measurement of neuronal signals, termed *neuronal integration.* The third uses formal *modelling approaches* to the fMRI signal. Below we review the methods and exemplar applications within each strategy, and illustrate their strengths and weaknesses for studying the effects of ageing.

### (a) Vascular detection using fMRI

The first class of approaches focuses on estimating vascular contributions to the BOLD signal using an independent MRI-based measurement that aims to capture individual variability in one or more of the physiological factors discussed in the previous section. An implicit assumption of these methods is that they explain variability in vascular signals (see figure 2), but not variability related to neural activity, such that they can be used to ‘adjust’ the BOLD signal without removing age-related neuronal changes. These approaches further fall into calibration and normalization methods. We focus here on task-evoked BOLD responses in voxel-wise imaging, but the principles can be applied to other forms of analysis.

#### (i) Normalization using baseline cerebral blood flow

Regional baseline CBF has for many years been measured with positron emission tomography (PET) [106] or MRI using tracer kinetic procedures [107]. However, safety concerns associated with tracers and the complexity of procedures have limited their application in BOLD studies of ageing. The predominant fMRI method for estimating resting CBF is based on endogenous contrast generated through perfusion of blood water into brain tissue [108]. The signal intensity is generated by applying a magnetic label to proton spins of the inflowing arterial blood water, termed arterial-spin labelling (ASL) [109]. In analogy to PET perfusion imaging, the ASL ‘tracer’ is the endogenous arterial blood water, where the magnetic label decays with T1 instead of radioactive decay. Deep understanding of the physiological basis of ASL and validation against radiotracer approaches [110–115] has established ASL as a robust and non-invasive technique to provide quantitative estimates of baseline CBF. An overview of ASL-variations and an agreement on its application have been discussed previously [116].

Resting state ASL studies of ageing support the presence of age-related atrophy-independent decreases in resting CBF throughout the cortex [117–119]. Some studies also suggest a nonlinear effect across the lifespan [120]. Interestingly, increases in regional CBF, in lateral and medial temporal lobe for example, have been observed with increasing age [118,121,122], which may reflect macro-vascular artefacts [123,124] owing to prolonged arterial transit time with ageing [125].

ASL studies support the proposal that age-related decline in baseline CBF reflects both cardiovascular and neurovascular impairment [119]. For example, age-related reduction in baseline CBF occurs in cortical regions typically associated with high vascular risk and genetic factors [126,127], and precedes brain atrophy [128–130]. However, recall that baseline brain perfusion is highly dependent on other physiological factors, and the difference in CBF may also reflect age bias in these factors, rather than baseline changes in CBF [131]. For example, baseline ASL may reflect spontaneous CO_2_ fluctuations, medication use, time of day, or levels of wakefulness [132]. Some influences are global and related to vascular tonus, while other local variations are the result of psychotropic effects on the brain. As an example, physical exercise, drinking coffee or smoking just before the perfusion measurement have substantial influence on both global and local quantification [133–136]. While this may be a drawback for absolute CBF quantification, it is an advantage for the use of ASL as a normalization technique, i.e., to control for multiple physiological factors in BOLD studies.

ASL has been broadly used to estimate baseline CBF, and has been used to try to rule out drug effects on vascular contributions to BOLD effects of disease or drug [137,138]. However, it is rarely used for a formal normalizing approach in evoked BOLD studies of age. This could be owing to the low signal-to-noise ratio of ASL, low spatial resolution and additional time needed to acquire baseline CBF, and a preference to integrate it within a BOLD fMRI acquisition (see §3a(iii) below). In one study, regional age-related differences in BOLD activation were shown to be mediated by baseline ASL-CBF, suggesting a substantial vascular contribution with regional specificity to the observed BOLD age differences [139]. In summary, the improvement in quality and application of baseline ASL-CBF measurements in recent years offers advantages over some of the other following approaches to control for age-related differences in physiological influences of BOLD signal.

#### (ii) Normalization using cerebrovascular reactivity

This approach differs from the baseline perfusion approach in that it relies on experimentally perturbed physiological states during the MRI scan. This physiological response, defined as cerebrovascular reactivity above, leads to changes in BOLD signal that are dominated by vascular factors (reflecting transient variations in physiological factors) in the absence of apparent changes in neuronal activity. In particular, estimation of cerebrovascular reactivity exploits the molecular mechanisms of CO_2_-induced vasodilation (discussed in §1b above), which can be used to model variability in physiological signals of evoked BOLD data. Fluctuations in arterial blood CO_2_ can take three forms of hypercapnia: CO_2_ administration, voluntary breathhold or naturally occurring fluctuations linked to respiration during a resting state fMRI acquisition (discussed in 3a(ii) and in [140]). As the CVR manipulations work under the assumption of no changes in the underlying neuronal activity and oxygen extraction (CMRO_2_) [64], the CVR manipulation takes the form:

$$\Delta \text{BOL}{\text{D}}_{\mathrm{CVR}}=M(1-f_{\mathrm{CVR}}^{\alpha -\beta }),$$

where *f* = CBF/CBF_0_ represents CBF signal normalized by its respective baseline value. The subscript CVR denotes the hypercapnia condition and the parameter *M* defines the maximum possible BOLD signal change for a brain region. The superscript parameters are determined empirically, but are well approximated as *α* ≈ 0.4 and *β* ≈ 1.5 [6]. Dividing the task-based BOLD response by the hypercapnia response yields a normalized BOLD response of the form:

$$\Delta \text{BOL}{\text{D}}_{N}=\frac{\left(1-f_{F}^{\alpha -\beta }m_{F}^{\beta }\right)}{\left(1-f_{\mathrm{CVR}}^{\alpha -\beta }\right)},$$

where *m* = CMRO_2_/CMRO_2,0_ represents CMRO_2_ signal normalized by its respective baseline value. The subscripts *N* and *F* denote the normalized BOLD response and the functional responses, respectively. Note that the *M* term cancels out in the normalized response, which precludes estimation of modulatory factors of *M*, such as magnetic field strength and baseline blood volume and oxygenation [141]. This normalization procedure entails the division of a functional contrast map by a CVR map, i.e. normalization at each voxel. However, including the hypercapnia response as a covariate in a voxel-level, general linear model (GLM), together with the functional BOLD response (e.g. when predicting behavioural or demographic measures) might provide a better approach [142]. It is worth noting that these operations assume a linear relationship between CBF and BOLD signal that holds across varying CO_2_-levels in arterial blood. However, this may not always be true [143], given claims of a nonlinear BOLD–CBF relationship [144] and a nonlinear response of vasculature to large CO_2_ and arterial pressure fluctuations [101,145]. This is further complicated by the ‘vascular steal’ phenomenon of flow diversion from regions of low to high cerebrovascular reactivity [146] and interactions between multiple physiological factors that increase with age [22]. While this warrants future research on modelling the nonlinear nature of the effects, the current approaches may lead to underestimation, rather than overestimation, and therefore still offer a partial solution to minimize vascular influences in evoked BOLD signal.

##### CO_2_-induced hypercapnia

An individual's hypercapnia response can be modulated by inhaling a special gas mixture inside the MRI scanner. Bandettini and Wong [141] were the first to demonstrate the utility of this technique for BOLD fMRI studies. (For a technical review and practicalities of this approach using various types of apparatus, see Liu *et al*. [140,147] and Germuska & Wise [148].) Regardless of the gas-delivery apparatus, accurate assessment of CVR relies on tracking the maximal concentration of CO_2_ in the exhaled air—so-called ‘end-tidal CO_2_’ (Et-CO_2_)—during breathing cycles with varying CO_2_ concentration in the inhaled gas (see in video format, [149]). The variation of Et-CO_2_ is tightly linked to changes in alveolar pressure of CO_2_ and fluctuations in arterial vasodilation, indicating the extent to which the vascular system is challenged. The analysis of CO_2_-induced hypercapnia data is conceptually similar to the task-evoked fMRI, where the temporally aligned Et-CO_2_ timecourse is included as the main regressor in a GLM to produce a cerebrovascular response (CVR_CO_2__) brain map. Early studies of CO_2_-induced cerebrovascular reactivity demonstrate a close regional overlap between voxels showing BOLD CO_2_ responses (BOLD-CVR_CO_2__ map) estimated from fMRI and voxels showing a cerebrovascular response (CBF-CVR_CO_2__ map) estimated from PET [150] and ASL [143,151,152]. Although this overlap has high reproducibility [153] across various field strengths and MRI sequences [154], BOLD-CVR_CO_2__ is more sensitive to basal CO_2_ fluctuations than CBF-CVR_CO_2__ [143].

BOLD-CVR_CO_2__ in grey matter declines with age [11,152,155–157]. The age effects on BOLD-CVR_CO_2__ are more prominent than those on baseline ASL-CBF [155,156] and exhibit distinct regional patterns [117], supporting the notion of age having independent effects on CVR and baseline CBF [118]. Interestingly, age-related BOLD-CVR_CO_2__ increases are found in white matter [157], which may reflect changes in the mechanical properties of the white matter: white matter in older adults becomes less densely packed owing to demyelination and axon loss, making it easier for blood to penetrate and vessels to dilate.

Combining BOLD-CVR_CO_2__ with evoked BOLD studies of ageing allows correction for regionally specific effects [11,158], which could lead to improved associations between BOLD estimates and outcomes of interest [159]. For example, age-related decreases in evoked BOLD responses in V1 and medial temporal lobe were abolished after correction, while age-related increases in bilateral frontal gyrus remained after correction. This suggests that many age-related differences found in fMRI studies reflect changes in vasodilation rather than in neuronal activity.

Unfortunately, such corrective methods have not been widely used, in part owing to impracticalities of large-scale studies, and tolerance by older adults and clinical populations [160,161]. Furthermore, a gas-induced hypercapnic challenge may not be neuronally neutral [162–165], e.g., given participants' awareness of the aversive challenge, and this effect on neural activity may differ with age [166]. In this case, correction by CVR_CO_2__ might obscure true task-related neural differences with age. Nonetheless, recent developments in the gas challenge procedure allow for estimation of multiple physiological parameters, including venous oxygenation and resting state functional connectivity (see [167]), which may improve the accuracy of corrections for vascular signals in BOLD fMRI studies.

##### Breath-hold-induced hypercapnia

An alternative way to modulate arterial CO_2_ in the absence of gas-delivery apparatus involves breathing challenges, where participants endogenously increase arterial CO_2_ by voluntarily holding their breath [168], which we term CVR_BH_ to indicate breath-holding [169]. BOLD-based CVR_BH_ demonstrates high correspondence with ASL-based CVR_BH_ [170,171], BOLD-CVR_CO_2__ ([172–174]; cf. [175]) and has excellent repeatability [176,177]. Improved CVR_BH_ estimation may be achieved using variations in the breath-hold procedure [178–181] and in analysis of the data [176,182,183].

Beyond its use to minimize inter-individual variability of physiological influences in BOLD studies of young adults [142,172,174,184–187], breath-holding has been used more commonly in ageing studies than other normalization approaches [172,188–194]. Riecker and colleagues showed that the age differences in BOLD response of sensorimotor regions during finger tapping were accompanied by differences in BOLD-CVR_BH_ [194], which was one of the first indications that evoked fMRI studies of ageing require careful interpretation of observed BOLD differences. Later studies extended these findings to other primary sensory regions, and corroborated the idea that age-related decline in evoked BOLD response to sensorimotor stimuli can be accounted for by age differences in CVR_BH_ [172,191]. Interestingly, age differences in BOLD signal in ‘higher-order’ cortical regions during cognitive tasks often remained after controlling for CVR_BH_, suggesting the relationship between BOLD signal, neural activity, vascular signal and age varies across brain regions [172,191]. More recent studies confirm that consideration of CVR_BH_ not only changes the pattern of regional age differences in evoked BOLD response [188,192,195], but also improves the strength of the relationship between BOLD responses and performance on the task [196].

However, while breath-holding may be more tolerable and has been employed in more ageing studies than gas-induced CVR, the compliance to the breath-holding procedure, lung capacity, inspiration and expiration ability of participants may decrease with their age [197]. Such biases affect data quality and reliability measures [175,179], highlighting the advantage of other less invasive (task-free) estimates of vascular components of BOLD time series.

##### Resting state fluctuation amplitudes

One such ‘task-free’ estimate of the vascular component of the BOLD signal is the intrinsic variability of the BOLD signal across time (after bandpass filtering to remove slow drifts in MRI signal and high frequency motion artefacts). This is known as resting state fluctuation amplitude (RSFA). Early studies demonstrate that RSFA reflects naturally occurring fluctuations in arterial CO_2_ induced by variations in cardiac rhythm and in respiratory rate and depth [198,199]. RSFA approximates the BOLD response to hypercapnic challenge and was proposed as a safe, scalable and robust cerebrovascular reactivity mapping technique [12,200]. As with other methods discussed above, the use of RSFA as a correction method for BOLD requires the assumption that age differences in RSFA reflect only vascular factors, rather than age-related differences in neural function. Although RSFA demonstrates high correspondence across brain regions and individuals when compared to baseline ASL-CBF [118,201], BOLD-CVR_BH_ and BOLD-CVR_CO_2__ [197,200,202], and in groups with compromised CVR [203,204], the effects of age on RSFA cannot be fully explained by these factors [118,201]. Therefore, without understanding the unexplained effects of age on RSFA in terms of neuronal versus vascular influences it would be dangerous to use RSFA as a normalization technique.

Recent evidence has improved our understanding of the origins of RSFA. For example, pulsatile effects could influence the BOLD signal in the proximity of large brain vessels and cerebrospinal fluid. In particular, the age-related increase in pulsatility deeper in microvasculature (see *Pulsatility* §2b(iii)) is likely to contribute to the RSFA signal, as recently recognized [12,96–100]. This could explain why Tsvetanov and colleagues [12] found that age differences in RSFA are either fully or partly mediated by heart rate variability. In contrast, these authors further found no evidence that neural variability (as measured by magnetoencephalography (MEG)) mediated the age effects of RSFA [12]; these findings were further supported by EEG-based neural estimates [205]. However, while age-related differences in RSFA may not reflect neuronal signals, the use of either somatic vascular measures or cerebrovascular measures explained only part of the age-related differences in RSFA. This leaves open the possibility that age-related differences in RSFA reflect joint contributions from cardiovascular and neurovascular factors, as in the case of BOLD signal fluctuations [206,207].

To resolve this ambiguity, we followed up our original study by considering the simultaneous assessment of the independent and shared effects of cardiovascular, cerebrovascular and neuronal effects on age-related differences in RSFA [118]. After controlling for either cardiovascular and neurovascular estimates alone, the residual variance in RSFA across individuals remained significantly associated with age, replicating the above findings. However, when controlling for both cardiovascular and cerebrovascular estimates, the residual variance in RSFA was no longer associated with age. This suggests that cardiovascular and cerebrovascular signals are together sufficient predictors of age-related differences in RSFA. In summary, while originally proposed to control for CVR [200], RSFA captures multiple vascular signals that are independently affected by age, and appears to be a valid method to correct for vascular factors in the BOLD signal, in order to better characterize effects of age on neural, and ultimately cognitive, function.

When RSFA is used to correct evoked BOLD data, the amplitude and spatial pattern of the normalized response are similar to that when using CVR_BH_ and CVR_CO_2__ [200]. Controlling for RSFA has been shown to minimize non-neural BOLD variability across individuals [186] in populations with impaired cardiovascular health [208,209], and improve estimation of evoked BOLD signals related to distinct neuronal mechanisms [210]. In studies of ageing, controlling for RSFA in evoked BOLD signal accounts for age-related differences in BOLD response in some sensory regions, comparable to findings from alternative normalization approaches [12,190,191,211]. Importantly, not all age differences disappear after controlling for RSFA—for example, the ipsilateral motor cortex overactivation in older adults remains, consistent with results from other approaches used to study ageing effects on the motor system [212–215].

Variations in the estimation of RSFA exist, which may be more sensitive to CVR relative to cardiovascular signals [216]. In addition, other means of RSFA-like estimates have been proposed to derive from non-resting cognitive states [217] or fixation-/resting-periods succeeding task periods [201]. Given that short periods of cognitive engagement have been shown to modulate the BOLD signal in a subsequent resting state scan [218,219], future studies are required to generalize RSFA findings to RSFA-like estimates derived from other types of fMRI acquisitions.

Other CVR-induced variations

The measures discussed above could be complemented with other physiological measures, such as total baseline venous oxygenation from phase contrast MRI [220], which may provide superior signal-to-noise ratio compared to ASL, or cerebral blood volume [114] with multiple physiologic parameters [221]. Their usefulness for estimating age-related differences in the vascular component of the BOLD signal remains to be demonstrated.

#### (iii) Calibration using concurrent fMRI

The use of so-called ‘calibrated fMRI’ has been possible for many years and can in theory control for differences in both baseline physiology and haemodynamic coupling across individuals and hence ages. The technique involves measuring blood flow, blood volume and venous oxygenation via normocapnic and hypercapnic/hyperoxic gas challenge during a concurrent measurement of BOLD and CBF [148,222,223]. As such, calibrated fMRI provides a measure of relative changes in CMRO_2_, which can be integrated with physiological models [224,225], to estimate quantitatively the absolute rate of cerebral metabolic oxygen consumption (CMRO_2_), i.e. oxidative metabolism, from the data. However, it has not seen widespread adoption, mainly because it is difficult to implement.

In theory, measurements of task-evoked oxidative metabolism provide quantitative estimates of CMRO_2_, which can help understand neuronal differences in oxidative metabolism across age groups even if they have differences in vascular health [10,158,226–228]. However, for at least some of this work, the motivation has been to distinguish the physiological components underlying BOLD in attempts to more narrowly isolate the age differences in CMRO_2_. For instance, Ances and colleagues [226] found age differences in M, vasodilatory capacity, as the principal difference between their young and old groups. Hutchison and colleagues [10], however, isolated the differences to the decoupling change in CBF relative to CMRO_2_, i.e. decoupling of CBF and CMRO_2_, implicating neurovascular impairment as an underlying factor of neural efficiency [229–231]. Measuring task-induced relative changes with the calibrated approach relies on several assumptions, including a constant coupling between cerebral blood flow and cerebral blood volume across individuals and brain regions [224]. This could lead to ambiguity of interpretation as the baseline may also be changing with age. Yet the CBF–CBV coupling seems to be regionally specific, and depend on disease stages [224], therefore requiring additional physiological measures [114,148]. These factors may explain the low frequency of use of such calibrated fMRI.

#### (iv) General remarks on normalization and calibrating techniques

Advancing age is associated with multiple alterations in cellular and structural vasculature, leading to multiple physiological changes that directly influence the BOLD signal. However, in contrast to the over 10 000 fMRI papers on ageing during the past 20 years, there are fewer than 100 papers addressing calibration and normalization techniques of fMRI-BOLD signal, and fewer than 20 independent studies of ageing that have applied these techniques. Future studies of ageing should consider the above correction methods, as we expand in §4.

A major issue is that nearly all the approaches reviewed above assume that vascular (age-related) factors have a linear influence on BOLD signal. However, there are nonlinear influences on the BOLD signal [145], which is rarely factored in analysis of BOLD data. Individual differences in vascular factors may occur singly, though more often in combination during ageing [22]. Moreover, these effects might be relatively independent of one another in early adulthood, but become increasingly coupled with advancing age. It is difficult to define which particular vascular factor might be primarily responsible for age-related changes and the degree and extent of their influence on BOLD signal. This is further complicated by the drugs and medication (e.g. used to normalize blood pressure) that are more often taken in old age. More research is needed to test which correction method (or combination of correction methods) can best correct for cerebrovascular influences to the BOLD signal in ageing studies.

### (b) Neuronal integration

The second class of approaches focuses on estimating neuronal contributions to the BOLD signal using independent measures of neural function. The advantages of such an integration approach, as opposed to working solely with measures of neural function, are discussed below. An implicit assumption of these approaches is that they explain variability in neural activity (figure 2), but not variability related to non-neuronal physiological signals. It is also important to note that such integration will work for age effects detected jointly by both modalities, but neural signals identified uniquely by either modality may remain undetected in the data.

Electroencephalography (EEG) and MEG (together M/EEG) are two widely used non-invasive techniques in neuroscience, and ageing. Although each technique provides important insights in isolation, there are advantages to integrating fMRI and M/EEG in a multimodal approach that is more powerful than each one alone [232]. We introduced fMRI as an indirect measure of neural activity with a temporal resolution of seconds, but with a spatial resolution of millimetres. M/EEG, on the other hand, directly measure millisecond electromagnetic activity from large populations of neurons, but at the cost of far worse spatial resolution, particularly for sources of that activity that are deep in the brain. Therefore, combining evidence from M/EEG and fMRI-based techniques can to some extent complement the inherent limitations within each individual imaging modality [14,232,233]. For example, M/EEG can be used to identify neuronal components and events beyond the temporal resolution of fMRI [234,235], while fMRI can be used to improve the spatial resolution of M/EEG signals [236,237].

The primary neural source of BOLD signal is synaptic activity in the grey matter, rather than spiking activity, as indicated by a closer relationship of the BOLD signal to local field potentials than multiunit activity recordings [238–240]. BOLD–M/EEG associations span multiple frequencies, although those in the gamma band appear most notable. These gamma oscillations (greater than 30 hz) are themselves too fast for BOLD to follow, but fluctuations in their power or amplitude envelopes typically fall in similar frequency range to the BOLD signal. Nonetheless, it is important to consider all neuronal frequencies together as a collective account of the fMRI signal, even if differential contributions are found across frequencies [241–244] and even if different frequencies are differently affected by ageing.

The main advantage MEG has over EEG is that the neuronal sources, though difficult to localize precisely, are better localized than with EEG. Thus while EEG has been used to study neurocognitive ageing for many years, MEG is making an increasing contribution, particularly in combination with formal models of neuronal circuits and their dynamics [245–249]. However, reports that combine task-based MEG with task-based fMRI are only starting to emerge [245], and the combination of MEG with calibrated fMRI in cognitive experiments [250] allows integration of MEG, BOLD and CBF responses to better study differences in neurovascular coupling with ageing.

One advantage that EEG has over MEG however is that it can be acquired simultaneously with fMRI, which is especially important for task-free states that are hard to replicate when EEG and fMRI are run separately [232]. EEG has been used to separate neural from vascular components of the BOLD signal [235] and decompose subcomponents of the haemodynamic response function [192,195,251]. One challenge for concurrent EEG–fMRI remains the strong magnetic interference in EEG signals [252], although this can be mostly overcome during data processing [147]. In the context of ageing, a study using concurrent EEG–fMRI reported a small number of age-related BOLD components that were associated with EEG [253], suggesting that the combination of both methods can better dissociate neural from non-neural signals than fMRI alone. In addition, there was a set of BOLD components that were not related to EEG components, and vice versa, and it remains unknown whether these components have a neuronal or non-neuronal origin. Since MEG and EEG have different sensitivities to different types of neuronal sources (depending on the orientation and depth of the underlying synaptic currents; [254,255]), it is advantageous to combine them too [237,256]. Indeed, in future work, EEG data could be acquired simultaneously with MEG and then simultaneously with fMRI, so as to provide a bridge between all three modalities.

There are other techniques, such as measures of glucose utilization [257], beta-amyloid burden [258], synaptic density [259], optical imaging [260], PET markers of neuroinflammation [261,262], MR spectroscopic measures of the neurotransmitters [263,264] and even non-invasive brain stimulation [265,266] that may further help understand the basis of BOLD signals, but are beyond the scope of this review.

### (c) fMRI modelling and signal decomposition

The third class of approaches focuses on formal modelling or statistical decomposition of the relative contribution of vascular and neuronal factors in the observed BOLD signal. Formal modelling approaches include linear models like the GLM, in which age-related variation can be captured by basis functions, as well as more complex, nonlinear, biophysical models that use differential equations that capture how neuronal events elicit variations in CBF, CBV and CVR and then ultimately changes in the BOLD signal [267,268]. Fitting these models to BOLD data results in estimates of various parameters that can then be related to age. Statistical decomposition approaches, on the other hand, are data-driven, such as principal component analysis (PCA) and independent component analysis (ICA). Both modelling and decomposition approaches can be useful even when there are no other measures of vascular or neuronal signals (just BOLD data). However, the application and interpretation of these approaches need to be treated with care: biophysical modelling for example typically operates within a high-dimensional space with highly covarying parameters (often requiring prior constraints on the parameters, based on other physiological knowledge), while decomposition techniques will optimize the selection of signals based on a specific statistical criterion; e.g. PCA optimizes for variance, while ICA optimizes for independence.

#### (i) The haemodynamic response function

The temporal evolution of the BOLD response to a brief burst of neuronal activity (an impulse response) is characterized as the *haemodynamic response function* (HRF)*.* The HRF typically peaks at 5–6 s, followed by an undershoot that lasts 20–30 s. The precise HRF shape varies across cortical regions, individuals and brain states. This variability is caused by variability in neurovascular coupling and cerebrovascular function, even in the presence of unchanged levels of neural activity [8,269]. One notable finding comes from Cohen and colleagues [53], who demonstrated that varying levels of CBF (induced by hypercapnia, normocapnia and hypocapnia) mediated the onset time, time-to-peak and amplitude of the HRF under the same visual stimulation. The HRF has also been shown to change with genetic, hormonal and other systemic fluctuations [270–272]. Regional variability in the HRF is partly dictated by the size of surrounding blood vessels, e.g. regions with larger draining veins have a more delayed HRF [273–275]. While some fMRI studies use a temporal basis set within the GLM to allow for variations in HRF shape across voxels/individuals [7,276], many use a single, ‘canonical’ HRF. In the latter case, any inferences about age differences in neuronal activity are complicated if there is a systematic age-related bias in the HRF [277]. For example, a 2-second mis-estimation in the latency of the HRF could (artificially) decrease the magnitude of the estimated neural response by 38% [273]. One potential way to address this would be to demonstrate the specificity of the findings to the condition of interest, but not other contrasts in the experiment.

Several studies have used multiple temporal basis functions with the GLM to capture age-related variations in the HRF shape, including approaches where estimation of the basis function parameters is jointly optimized with estimation of brain activity [278,279]. However, the results have been mixed (see [280]), which might reflect variability across studies in the tasks, the use of relatively small sample sizes and biased selection of participants (particularly when older volunteers are more healthy than average). The recent study by West *et al*. [280] addressed these problems by using a large, population-derived cohort called Cam-CAN, in which a simple sensorimotor task was optimized for detection of HRF shape [281]. This study found extensive effects of age on the HRF, particularly its latency, in many brain regions, despite the fact that there were no performance differences between young and old adults (although latencies of neuronal responses were not directly measured).

#### (ii) Dynamic causal modelling

Dynamic causal modelling (DCM) is a model-based approach to studying brain connectivity [282], which includes a biophysical model of the BOLD response [267,268]. DCM uses a Bayesian framework to simultaneously estimate parameters capturing neural activity (and connectivity) and parameters capturing the vascular mapping of that activity to the BOLD response. The neural activity can either be defined by experimental manipulations [282] or by assumptions about the endogenous fluctuations that occur in task-free states like rest [283]. Importantly, the simultaneous optimization of neuronal and vascular models (unlike in GLM approaches above) means that differences in the estimated vascular parameters (e.g. owing to age) are, in theory, uncontaminated by any differences in neuronal parameters. However, the model can be under-determined (more degrees of freedom in the model than in the data), requiring strong priors on some of the parameters to regularize the models [284]. These include priors based on physiological data from previous studies, or shrinkage priors that require strong evidence in order for posterior estimates to differ from their prior expectation. The model optimization is made with reference to (log)-model evidence, which accounts for both model accuracy and model complexity.

We applied DCM to resting-state data from 635 adults aged 18–88 in the CamCAN dataset [15]. A notable finding was that neural and haemodynamic parameters were independent predictors of age, supporting the hypothesis of separable mechanisms leading to age alterations in neural and vascular function. Furthermore, the neural (connectivity) parameters were related to cognitive ability, and this relationship was moderated by age, demonstrating the behavioural relevance of this approach to neurocognitive ageing. Interestingly, the same relationship to cognitive ability was not observed with traditional (correlational) analysis of BOLD functional connectivity, which confounds neural and vascular components of the BOLD signal. These findings motivate the use of modelling techniques like DCM to separate neural and vascular components of the BOLD signal.

#### (iii) Independent component analysis

ICA is a data-driven approach to extract signals (components) that are maximally independent across a dimension, such as across space when applied to fMRI images. McKeown and colleagues were some of the first to apply spatial ICA to fMRI BOLD data under the assumption that signal from each voxel represents a linear mixture of source signals [285]. Each ICA component consists of a spatial pattern across voxels associated with a common BOLD timecourse. These components are often dominated by neural or non-neural signals [286], and their spatial distribution (and/or power spectrum) can sometimes be used to identify vascular components (e.g. around the Circle of Willis) or other noise sources (like motion artefacts, which often appear around the edge of the brain). Another way to separate BOLD from non-BOLD components is to combine ICA with multi-echo fMRI: only ICA components dominated by BOLD signal should show a linear dependency on echo-time (TE) [287].

Most studies use ICA to extract functional networks from task-free fMRI data [15,288,289] or structured sources of signal from morphological, vascular and neuroinflammatory measures [12,262]. In task-based BOLD studies, the ICA approach offers multiple advantages over the traditional GLM approach [290]. It can separate and remove non-neuronal signals for improving the sensitivity of subsequent task-based GLM analysis [289,291]. ICA can also identify task-based BOLD changes in a model-free manner that can minimize sensitivity to variation in the HRF shape [292,293]. Finally, ICA can dissociate between multiple concurrent processes associated with common regions under varying cognitive states [294]. Despite these advantages, future studies need to benchmark the efficiency of ICA to control for age differences in neurovascular coupling against other data-driven decomposition approaches [295] and normalization methods of task-based BOLD.

## 4. Towards neuro–vascular integration

The previous sections highlighted methodological approaches to addressing age-related changes in cerebrovascular function at the cellular, structural and physiological levels in the context of BOLD fMRI and neurocognitive ageing. This methodological emphasis reflects the currently dominant aim in neurocognitive ageing: to relate cognitive changes to changes in brain activity, such that changes in vascular components are confounds of no interest. Here we argue that, rather than ignoring or correcting for such confounds, we need a better understanding of the neurovascular contribution to neurocognitive ageing, and to formally integrate vascular changes into models of successful ageing.

Vascular mechanisms in the brain do not simply control blood flow to support the metabolic needs of neurons, but lead to complex neurovascular interactions that shape neuronal function in health and disease [16,25,94]. Microvascular changes lead to lacunar infarcts, cortical and subcortical microinfarcts, microbleeds and diffuse white matter disintegration, which involves myelin loss and axonal abnormalities [296]; all of which potentially impact cognition. Brain areas in relatively sparse regions of the microvascular network, including deeper structures and white matter, are particularly vulnerable, predicting specificity in resulting cognitive deficits [94]. Age-related deficits in cognitive function have been linked to cardiovascular risk factors [297], white matter hyperintensities [298], increased pulsatility [94] and neurovascular coupling impairment [16], which may act through independent pathways [299]. Furthermore, improvement in cognitive function has been linked to increase in cardiovascular health [300]. These findings suggest that the components of cerebrovascular function are not simply confounders that obscure brain–behaviour relationships, but are synergistic factors that facilitate maintenance and improvement of cognitive function across the lifespan [16,94]. Therefore, formal integration of neurovascular knowledge provides an opportunity for a more comprehensive understanding of successful cognitive function in ageing.

Current models of neurovascular ageing [25,301] provide an array of biological pathways leading to global brain tissue loss/atrophy and cognitive deficits, mainly in age-related neurodegeneration. However, such models are suboptimal for characterizing healthy and successful ageing, where cognitive function is maintained in the presence of brain atrophy [302]. In addition, the link between age-related changes in brain tissue and cognition is surprisingly weak, and it has proven difficult to establish region-by-region correlations between brain structure and cognitive function [303]. Moreover, not all cognitive abilities decline with age, nor do all older adults show cognitive decline at the same rate. Studying the effect of neurovascular ageing on brain atrophy or global cognitive decline on its own is insufficient for understanding the complex pattern of cognitive diversity and increasing individual variability in healthy ageing [304,305].

In the field of neurocognitive ageing, the advent of functional imaging and its early emphasis on functional segregation bolstered the idea that the brain can flexibly respond to age or tissue loss, by recruiting additional brain regions to support cognitive functions [306]. Many theories of cognitive ageing have since emerged [306], some proposing that the recruitment of additional brain regions improves performance, while others suggest it can impede performance [307]. Currently, there are three general models of successful ageing in terms of sustained cognitive performance: *maintenance, reorganization* and *reserve* [308], which are not necessarily fully compatible. In our view, these models demand more sophisticated interpretation of BOLD fMRI [309] through the integration of neurovascular ageing [12,15]. It is important to ask whether and how multiple cerebrovascular components (in models of neurovascular ageing) independently and synergistically explain multiple profiles of neural function leading to cognitive diversity in ageing [289].

We propose that one should consider cerebrovascular function as an additional predictor in the modelling of brain–behaviour relationships, rather than simply a normalization or confounding variable. This will provide a more complete interpretation of the unique and shared contributions to brain–behaviour relationships. For example, the shared variance in task performance explained by vascular and neural signals indicates the presence of a common underlying factor. Conversely, the unique variance explained by neural signals suggests that the effects are beyond differences in cerebrovascular function. Finally, unique variance explained by vascular signals may indicate that the neuronal estimates are insufficient to capture all behavioural variability and an improved definition of the neuronal estimates should be reconsidered. These scenarios are plausible in isolation or in combination with one another, but importantly their consideration provides an empirical motivation to understand what determines cognitive diversity in ageing. Furthermore, modelling and reporting the effects of cerebrovascular function on the brain–behaviour relationship is in the spirit of maximizing internal validity [310], avoiding pitfalls of modular analysis [311], transparent reporting of results, facilitation of replication and interpretation of findings within the context of the limitations of the research methodology providing the signals of interest.

In summary, we argue for the integration of neurovascular and neurocognitive research on biological, theoretical, methodological and analytical grounds. We propose that future research should focus on the interplay of vascular and neural factors for maintaining mental health across the lifespan (i.e. successful ageing) using a multi-modal, integrative approach. Integration of neurovascular and neurocognitive ageing could provide new insights into the fundamental mechanisms that regulate brain health and mental well-being. Importantly, it will determine the extent to which these factors relate to neural function, relate to cognitive performance, and are associated with individual differences in lifestyle, demography, genetics and health. This will provide a bridge between modifiable risk and protective factors, neurovascular function and cognitive ability across the healthy adult lifespan.

## 5. Conclusion

With recent advances in fMRI BOLD imaging, much has been learned about the effects of age on neurovascular and neurocognitive function. It is clear that neurovascular and neuronal signals both contribute to fMRI BOLD signal, and that their interaction affects the interpretations one can draw about neurocognitive ageing. To understand the effect of ageing on brain function, a variety of techniques have been developed and validated that separate vascular from neuronal signals in BOLD-fMRI data. However, only a small fraction of fMRI studies of ageing have adopted such approaches in their analysis. We argue on biological, theoretical and analytical grounds for a better understanding of their relative contributions to fMRI. Vascular and neuronal contributions can be formally integrated in models of successful ageing, avoiding common misinterpretations of fMRI and complementing the limitations within individual modalities. Only by first understanding these mechanisms and their interactions can we subsequently address a major challenge that pervades neurovascular and neurocognitive ageing: to characterize the effects of healthy and pathological ageing at the level of vascular and neuronal network structures of the human brain across the lifespan.

## Data accessibility

This article has no additional data.
